# Plastic adjustments of biparental care behavior across embryonic development under elevated temperature in a marine ectotherm

**DOI:** 10.1002/ece3.7902

**Published:** 2021-07-29

**Authors:** Davide Spatafora, Gloria Massamba N'Siala, Federico Quattrocchi, Marco Milazzo, Piero Calosi

**Affiliations:** ^1^ Department of Earth and Marine Sciences (DiSTeM) University of Palermo Palermo Italy; ^2^ Département de Biologie, Chimie et Géographie Université du Québec à Rimouski Rimouski QC Canada; ^3^ Centre d'Écologie Fonctionnelle et Évolutive (CEFE‐CNRS) UMR 5175 Montpellier Cedex 5 France; ^4^ Institute for Marine Biological Resources and Biotechnology (IRBIM) National Research Council CNR Mazara del Vallo (TP) Italy

**Keywords:** behavioral plasticity, brood size, global warming, hatching success, invertebrates, parental investment

## Abstract

Phenotypic plasticity in parental care investment allows organisms to promptly respond to rapid environmental changes by potentially benefiting offspring survival and thus parental fitness. To date, a knowledge gap exists on whether plasticity in parental care behaviors can mediate responses to climate change in marine ectotherms. Here, we assessed the plasticity of parental care investment under elevated temperatures in a gonochoric marine annelid with biparental care, *Ophryotrocha labronica*, and investigated its role in maintaining the reproductive success of this species in a warming ocean. We measured the time individuals spent carrying out parental care activities across three phases of embryonic development, as well as the hatching success of the offspring as a proxy for reproductive success, at control (24℃) and elevated (27℃) temperature conditions. Under elevated temperature, we observed: (a) a significant decrease in total parental care activity, underpinned by a decreased in male and simultaneous parental care activity, in the late stage of embryonic development; and (b) a reduction in hatching success that was however not significantly related to changes in parental care activity levels. These findings, along with the observed unaltered somatic growth of parents and decreased brood size, suggest that potential cost‐benefit trade‐offs between offspring survival (i.e., immediate fitness) and parents' somatic condition (i.e., longer‐term fitness potential) may occur under ongoing ocean warming. Finally, our results suggest that plasticity in parental care behavior is a mechanism able to partially mitigate the negative effects of temperature‐dependent impacts.

## Graphical abstract

In this study, we assessed the plasticity of parental care investment under elevated temperatures in a gonochoric marine annelid with biparental care, *Ophryotrocha labronica*, and investigated its role in maintaining the reproductive success of this species in a warming ocean. These findings, along with the observed unaltered somatic growth of parents and decreased brood size, suggest that potential cost‐benefit trade‐offs between offspring survival (i.e., immediate fitness) and parents' somatic condition (i.e., longer‐term fitness potential) may occur under ongoing ocean warming. Finally, our results suggest that plasticity in parental care behavior is a mechanism able to partially mitigate the negative effects of temperature‐dependent impacts.

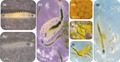

## 1 INTRODUCTION

Environmental temperature has ubiquitous effects on all aspects of organismal biology (Angilletta, 2009; Hochachka & Somero, 2002). This is particularly true for ectothermic animals, whose body temperature conforms to that of the surrounding environment and depends mainly upon external heat sources (Abram et al., 2017). In these organisms, the relationship between thermosensitivity and thermoregulatory capacity in variable environments governs the evolution of a wide range of behavioral, physiological, and life‐history traits, finally determining their overall fitness (Abram et al., 2017; Munday et al., 2009; Przeslawski et al., 2008). Phenotypic plasticity, that is, the capacity of a given genotype to produce a range of phenotypes under varying environmental conditions, is a key mechanism that allows ectotherms to cope with rapid thermal changes (Schlichting & Pigliucci, 1998). Depending on the effect of plasticity on individual fitness, plasticity can be defined as adaptive, if it improves a genotype's fitness when environmental conditions change, or neutral, if fitness is not affected (Ghalambor et al., 2007). Alternatively, plasticity can even be maladaptive if its expression decreases fitness (Schlichting & Pigliucci, 1998). Most research on thermal plasticity has been focused on physiological and life‐history traits, with only more recently an increasing number of studies considering the importance of behavioral traits for terrestrial and aquatic ectotherms (e.g., Abram et al., 2017; Huey et al., 2012; Nagelkerken & Munday, 2016). An even wider knowledge gap exists for marine organisms' ability to adjust specific fitness‐related behavioral responses when submitted to a thermal change. This paucity of information is particularly evident for parental care activities (Brante et al., 2003; Dick et al., 1998; Hopkins et al., 2011).

In species exhibiting parental care, variation in temperature conditions—far from their optimal thermal range—may alter the energetic investment required by parents to effectively perform such activity (Johnston & Bennett, 1996). As a result, parents may incur trade‐offs between behavioral and physiological processes, and thus between parental care and cell repair, homeostasis, feeding, and growth, which can ultimately affect organismal fitness (Ardia et al., 2009; Roff, 2002; Stearns, 1992). For example, a greater metabolic demand due to increased temperature can cause parents to devote less energy to parental care activities in order to favor self‐maintenance (e.g., cell repair and mass loss avoidance), thus enhancing their chances of survival and future breeding attempts (Wiley & Ridley, 2016). Alternatively, the maintenance of parental care and reproductive performance at a higher temperature may divert resources away from somatic maintenance (e.g., growth) (Donelson et al., 2010), with possible consequences for survival and life span fitness (Edward & Chapman, 2011). Changes in the amount of energy invested by parents caring for eggs and self‐maintenance due to intrinsic (e.g., age and health) and extrinsic factors (e.g., environmental conditions and predation) could also be a strategy adopted by species to favor future reproduction at the expense of current reproduction, as early postulated by *William's principle* (1966) and later supported by Carlisle (1982). Optimal parental behavior can also be indirectly affected *via* temperature‐dependent changes in embryos' development rate, size, and number (Angilletta et al., 2006; St Mary et al., 2004). Commonly, variation in clutch size has been shown to affect the amount of energy invested in parental care activity in fish (Coleman et al., 1985; Van Iersel, 1953) and invertebrates (Fernández & Brante, 2003; Rauter & Moore, 2004; Smiseth & Moore, 2004). Larger broods require a greater parental care investment (e.g., by fanning) to guarantee embryo development, likely because the lower surface/volume ratio of egg masses may cause a lower rate of oxygen diffusion especially in their center (Fernández et al., 2002). In addition to this, the increasing metabolic needs of embryos across developmental stages may alter the amount of energy/time allocated by parents for care activities (Baeza & Fernández, 2002; Dick et al., 1998; Green & McCormick, 2005).

Thermal changes can also have an asymmetric effect on parental care investment in iteroparous species with biparental care, due to differential impacts on caregiving timing and duration provided by each sex (AlRashidi et al., 2010; Vincze et al., 2017). Several theoretical models have been proposed to explain the conflict that occurs in biparental care regarding the level of investment that each parent provides (Houston, 1985; McNamara et al., 1999, 2003). According to one early model, also known as the “no negotiation model”, if one parent provides significantly less care due to a change in the environment (e.g., rising temperature) or due to changes in life‐history traits (e.g., brood size), the other partner may modify its effort independently of the effort adopted by the first parent (Houston, 1985). Inversely, as predicted by more recent models (i.e., “the negotiation models”), one parent may adjust its level of parental investment in relation to the decrease in parental care provided by its partner (e.g., Johnstone & Hinde, 2006; McNamara et al., 1999, 2003). Under such circumstances, the partner may have different options: (a) to abandon altogether the care of the offspring in favor of future longer‐term reproductive opportunities; (b) to reduce its parental care effort; or (c) to increase its parental care effort (Johnstone & Hinde, 2006; McNamara et al., 2003). To date, despite that numerous factors are known to affect biparental care patterns, such as mating system, developmental mode, and brood size (Houston & McNamara, 2002; Olson et al., 2008), the effect of rising temperatures on parental investment in species exhibiting biparental care remains poorly understood or completely overlooked, especially when concerning aquatic invertebrates.

In this study, we assessed the role of behavioral plasticity in mediating, or exacerbating, climate‐related impacts on organismal fitness using the marine annelid *Ophryotrocha labronica* (Eunicida, Dorvilleidae, La Greca & Bacci, 1962). *Ophryotrocha labronica* (max length = 4 mm) is a gonochoric species occurring in a variety of temporally and spatially fluctuating coastal habitats across the globe (Simonini et al., 2009). Females reproduce several times over an extended breeding period (defined as semicontinuous reproduction), spanning approximately between 83 and 16.5 days at 14.5 and 28℃, respectively (Åkesson, 1976). Females lay their eggs in characteristic tubular masses after a period of courtship with a male (Prevedelli & Simonini, 2001). Immediately before spawning, the couple move side by side emitting a loose jelly into which eggs and spermatozoa, which are almost immotile, are extruded; this behavior being known as pseudocopulation (Lorenzi et al., 2019; Paxton & Akesson, 2010). The tubular egg masses are formed before the surfaces of the egg mass harden. When individuals are isolated into pairs, *O. labronica* provide biparental cares to ensure the cleanliness and oxygenation of the eggs mass (Paxton & Åkesson, 2007). However, at higher densities, males can mate with multiple females, abandoning their partner at any time after the fertilization of one mass of eggs to breed with another female, ending up caring for only one of the egg masses fertilized (Picchi & Lorenzi, 2019; Sella & Bona, 1993). In both cases, females are considered the main caregivers and are constrained to parental care duties, while males can adjust their parental care effort at different densities to maximize mating opportunities (Picchi & Lorenzi, 2019). Parental care is necessary for the survival of the brood, as exemplified by the observation that eggs usually degenerate if parents are removed before embryos are completely developed (Paxton & Åkesson, 2007). Parental care enhances oxygenation of eggs and consists of active movements of the parents' bodies in close contact with the outer or internal surface of the tubular mass (Paxton & Åkesson, 2007). In addition, parents periodically clean the surface of the eggs mass with grazing‐like movements of their jaws, thought to prevent the proliferation of fungi, protozoans, and bacteria (Paxton & Åkesson, 2007; Sella, 1991). Parental care is provided until the embryos break free of the egg mass casing (Paxton & Åkesson, 2007), and its duration depends on the eggs' developmental time, which generally decreases under increasing temperatures, approximately between 3 and 9 days at 30 and 18℃, respectively (Åkesson, 1976; Massamba‐N'Siala unpublished data).

To achieve our goal, we first investigated the occurrence of changes in parental care in response to elevated temperatures in this marine annelid and then assessed whether thermal plasticity contributes to maintaining individuals' reproductive success. In particular, we exposed independent groups of *O. labronica* parents together with their spawned egg masses to control (24℃) and elevated (+3℃, RCP 8.5, IPCC, 2014) temperature conditions, and measured the amount of time spent by parents (individually and together) carrying out parental care activities. Then, we assessed whether variation in the time dedicated to parental care affected offspring hatching success, which was used as a proxy for parental fitness. Temperature is a major abiotic factor triggering plastic responses in *O. labronica* (Åkesson, 1976; Chakravarti et al., 2016; Gibbin, Chakravarti, et al., 2017; Gibbin et al., 2017; Jarrold et al., 2019; Massamba‐N'Siala et al., 2012, 2014; Prevedelli & Simonini, 2001). In this species, increasing temperatures induce physiological adjustments that underlie higher growth and reproductive rates, as well as reduced developmental times, age to sexual maturity, fecundity per reproductive events (brood size), and life span (Massamba‐N'Siala et al., 2012; Prevedelli & Simonini, 2001). Living at a greater pace of life may divert energy from parental care behaviors, which consist of energetically demanding activities (e.g., Baeza & Fernández, 2002; Green & McCormick, 2005). As a consequence, we expect parents to decrease the time spent to care for the offspring in favor of their self‐maintenance, with negative implications for the reproductive success for the specific breeding event. This decrease in parental care investment may also be favored by a reduction in brood size expected under increased temperature (e.g., Fernández et al., 2000).

In addition, to more accurately characterize the role of each parent in caring for the brood and assess whether their parental investment is differently affected by elevated temperature, we compared the time spent separately by each parent, as well as simultaneously, in taking care of the egg mass at the two temperature conditions tested. Based on the previous observation on sex‐specific behavioral patterns in *O. labronica* and specifically that males are less strictly bounded by parental duties (Kokko & Jennions, 2012; Picchi & Lorenzi, 2019), we expect that parental care activities will be more likely reduced or completely dropped by the male when compared to the female. Under these conditions, the decline in the male investment of caring for the eggs may leave the female with two main options: (a) maintaining her parental care effort in favor of her short‐term reproductive success, but with potential costs for her self‐maintenance or (b) reducing her investment in parental care to the benefit of her self‐maintenance, but at the detriment of her short‐term reproductive success.

## 2 MATERIALS AND METHODS

### 2.1 Specimens' collection and maintenance

*Ophryotrocha labronica* specimens used in this study are descendants of approx. 60 indiv. collected in Gela harbor (Sicily, Italy: 37°040N. 14°130E) as described by Massamba‐N'Siala et al. (2011), and then transferred to the Marine Eco‐Evolutionary Physiology laboratory at the University of Québec in Rimouski (QC, Canada). Individuals were divided into four glass bowls (70 mm diam., 30 mm height) and reared for approximately six generations in artificial seawater (Aquarium Sea Salt Mixture, Instant Ocean^®^, Blacksburg, VA, USA) at constant temperature (24 ± 0.5℃; mean ± *SD*), salinity (35 ± 2), pH (8.05 ± 0.1 units), and 12:12 light:dark photoperiod.

### 2.2 Experimental setup and design

**FIGURE 1:**
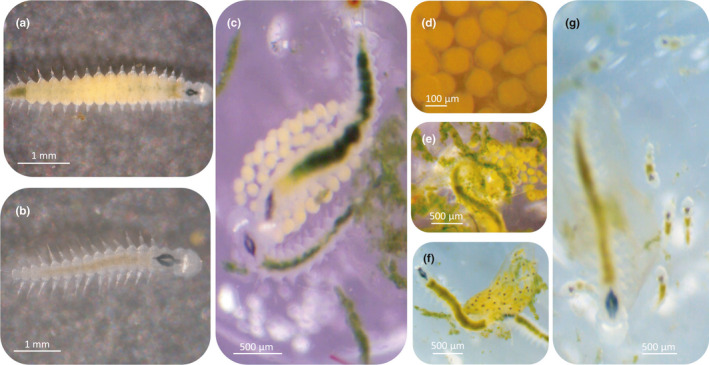
Adult female (a) and male (b) of *Ophryotrocha labronica* in dorsal view and during parental care activities (c). The stages that identified the start and end of the three phases of embryonic development considered in our study are also shown: phase 1 (d), phase 2 (e), phase 3 (f), and the hatching moments (g, end of phase 3)

To assess changes in *O. labronica* parental care behavior in response to elevated temperatures, sexually mature females and males (Figure 1a,b) were first randomly selected from the laboratory cultures to form 48 pairs (F0 generation), which were kept at the same conditions of previous exposure. Each pair was placed in one of the wells (34 mm diam., 20 mm height) of a 6‐well culture plate (Costar, VWR, Radnor, PA, USA) until the first egg mass production. When F1 individuals reached sexual maturity, 34 pairs were formed by crossing sexually matured males and females randomly chosen from different broods in order to avoid inbreeding. Each pair was isolated in one removable well and randomly assigned to one of two temperature conditions (17 pairs per condition): control (24℃) and elevated (27℃) temperature. The former temperature condition represents an average summer temperature (June–September) experienced by this species in the location where individuals were originally collected (Massamba‐N'Siala et al., pers. comm.), while the latter temperature condition represented a mean +3℃ of temperature increase expected by the end of the 21st century scenarios following the Representative Concentration Pathway (RCP) 8.5 of the Intergovernmental Panel on Climate Change (IPCC, 2014). The elevated temperature condition was reached from control conditions progressively (1℃/hr) (Massamba‐N'Siala et al., 2012) using a temperature incubator (MLR‐352H‐PA, Panasonic Healthcare Co. Ltd, Tokyo, Japan). Stable thermal conditions and 12 light:12 dark regimes were achieved by placing the culture plates in two incubators. Each plate was kept on separate shelves and cyclically moved to another shelf to remove the effect of the position in the incubator on our observations. To reduce evaporation, plates were covered with a breathable seal (AeraSeal, Alpha Laboratories Ltd, Eastleigh, UK). Throughout the experiment, individuals were daily fed ad libitum with minced spinach (Massamba‐N'Siala et al., 2012) to avoid food‐limiting conditions, which can affect parental care behavior (Arcese & Smith, 1988; Carlisle, 1982). Water changes were performed daily to prevent undesired fermentations and the accumulation of excreta, while maintaining stable oxygen levels (always >70%).

### 2.3 Determination of parental care activity

Video recording for parental care activities was performed with a digital camera (14 MP, Omax, Bucheon, South Korea) mounted on a light microscope (MS5, Leica, St. Gallen, Switzerland). During video recording, temperature conditions were maintained constant by immersing the experimental plate inside a water bath heated by two aquarium heaters (100 W Hydor, Sacramento, CA, USA). To ensure homogenous heat distribution, a submersible water pump (Koralia Nano 900, Hydor, Sacramento, CA, USA) was placed inside each water bath. Temperature was recorded continuously using a high accuracy J/K input thermocouple thermometer (HH802U, OMEGA, Laval, QC, Canada, ±0.1℃), while salinity was checked before and after video recording with a refractometer (DD H_2_Ocean, MOPS, Hamilton, ON, Canada, ±1.0 unit).

F1 pairs were checked several times on a daily basis. Whenever a female laid her first egg mass (Figure 1c), the well with the pair was moved in the system for video recording of parental care. Since the time frame of parental activities could change depending on the temperature condition, we divided the period of egg development (from spawning to hatching) into three phases representing specific stages of embryo development comparable between temperature conditions (Figure 1d‐f). We referred to Phase 1 (Figure 1d) as the time between the deposition of the egg mass and the first emergence of jaws in the embryos (Paxton, 2004). During this period, eggs had a roundish shape and a homogeneous yellow color. Phase 2 (Figure 1e) was defined as the time between the end of Phase 1 and the embryos' full body development. During this time, embryos changed from an elongated egg shape to the final shape observed in hatchlings. At the end of this phase, embryos started to actively move within the brood pouch starting Phase 3 (Figure 1f). This last phase ended when the larvae hatched, that is, when they broke free out of the envelope that protected them during the entire duration of development (Figure 1g) (Oyarzun & Strathmann, 2011).

*Ophryotrocha labronica* parental care behavior was assessed using a standard continuous focal sampling procedure (Martin & Bateson, 1993). We grouped into a single category of parental care activity all the behaviors identified by Paxton and Akesson (2010) (Table 1). Parental care activities were recorded for 30 min every 3 hr until the end of Phase 3, specifically during an average time of 6 days at 24℃ and 4 days at 27℃. Then, for each pair, we randomly selected one video corresponding to each of the three developmental phases previously identified, thus obtaining 1,800 s of recording for each phase that was used to monitor parental care behaviors. To explore whether temperature affected how parental investment was divided between sexes, we measured the individual contribution of each sex to parental care activity, which was defined as the proportion of time spent by parents performing parental care activity alone with their body in close contact with the egg mass (Picchi & Lorenzi., 2019). These time variables were defined as TF for the female and TM for the male. In addition, we measured the proportion time spent simultaneously by both parents caring for the eggs (defined as TS) and the cumulative contribution of TF, TM, and TS, defined as the proportion of total time for parental care activity (TT).

**TABLE 1:** Description of all parental care behaviors in *Ophryotrocha labronica*

Parental care behaviors	Cleaning and oxygenation by scratching or brushing the body on the eggs mass
	Cleaning the egg mass from debrides with jaw movements
	Parents in close contact with the eggs accompanied or not by clear peristaltic contractions

### 2.4 Determination of reproductive success and life‐history traits

Hatching success was measured as the number of juveniles that hatched successfully over the total number of eggs spawned. The count was performed by singularly moving each hatchling from the well to another well using a Pasteur pipette. Three life‐history traits were also considered to help control for other factors potentially influencing parental care investment: brood size, body size, and growth rate of both male and female. The number of eggs spawned by a female was used as proxy for brood size (*N* = 34), which is known to affect parental care behaviors (Rauter & Moore, 2004). Specifically, digital photographs of each egg mass were taken at first deposition using the digital camera mounted on the microscope, and the number of eggs was counted using the software ImageJ (Schneider et al., 2012). Given that parental care activity is mainly carried out through active movements of the parents' body over the eggs mass, we also measured female and male body size by counting the number of chaetigers (metameric segments bearing bristles) at the time of spawning and hatching of the larvae (Massamba‐N'Siala et al., 2012). This trait is known to be sensitive to thermal variations in *O. labronica* (Massamba‐N'Siala et al., 2012), and it is commonly positively correlated with brood size in females (Berglund, 1991). Finally, we measured parents' growth rate as the number of chaetigers added *per* day from the day females produced the egg mass to the end of parental care activity. Temperature‐dependent changes in growth rate are expected in *O. labronica* (Massamba‐N'Siala et al., 2012) and may divert energy away from parental care functions (Stearns, 1992).

### 2.5 Statistical analyses

#### 2.5.1 Effect of temperature and brood size on parental care activity

A set of preliminary analyses were performed to explore the effects of (a) seawater temperature on brood size, growth rate, and body size of parents and of (b) parental growth rate and brood size on the proportional time of parental care (see statistical analyses and results in Appendix S1 and S2). Only brood size significantly decreased at the elevated temperature (Table S1; Figure S1 in Appendix S1) and showed a positive relation with total time for parental care activity (TT): binomial generalized mixed model (B‐GLMM); Appendix S2, Table S3a and Figure S2. Therefore, to separate the effect of brood size from the effect of temperature on parental care behaviors, we calculated the “residual index” (Jakob et al., 1996) by extracting the regression residuals from the previous B‐GLMM between the proportion of TT and brood size, which represented the times of parental care activity controlled for brood size. We then assessed the effects of temperature (“Temp”—fixed factor with two levels: 24 and 27℃), embryo developmental phase (“Phase”—fixed factor with three levels: Phase 1, 2, 3), and their interaction on the “residual index” using a generalized least squares model (GLS; nlme package) (Pinheiro & Bates, 2000). GLS was used since no significant differences were found when comparing it with the linear mixed model (LMM) considering “Pairs” as random factor (likelihood ratio test LRT = 8.93e^−08^, *p* = 1.00). Female and male body size was not included in all final analyses because its effect was always found not to be significant (*p* > .05). Pairwise comparisons among least squares means for levels of factors were performed with Tukey's test by using the “lsmeans” package (Lenth, 2016). All analyses were performed using the R software version 3.3.0 (R Core Team, 2016).

#### 2.5.2 Effect of temperature and parental care activity on reproductive success

The effect of “Temp”, Total TT (used as continuous covariate and measured as the sum of TT measured at each phase: i.e., Total TT = TT(Phase 1) + TT(Phase 2) + TT(Phase 3)), and their interaction, on hatching success was analyzed with a Poisson distribution generalized linear model tests (P‐GLM). Brood size was used as an offset variable to scale the model because the quantification of hatching success was based on the total number of eggs spawned.

#### 2.5.3 Effect of temperature on the sex‐related division of parental care

Given that the proportion of TF, TM, and TS decreased significantly with the reduction in the brood size (Appendix S2, Table S3b‐d), we tested the effect of temperature on these descriptors by taking into account the effect of brood size, using the same procedure adopted for the proportion of TT. Specifically, we extracted the regression residuals from B‐GLMMs between each descriptor and brood size. The term “Pairs” was initially included as a random factor, but it was never significant (TF: LRT = 8.93e^−08^, *p* = 1.00; TM: LRT = 8.93e^−08^, *p* = 1.00; TS: LRT = 8.93e^−08^, *p* = 1.00). Thus, we used three GLS models, one for each descriptor's residual index, to test for the effect of the factors “Temp”, “Phase”, and their interactions on the proportion of TF, TM, and S. Post hoc pairwise comparisons using Tukey's test (“lsmeans” package) were also performed to assess the significant interaction between levels of factors.

## 3 RESULTS

### 3.1 Effect of temperature on parental care activity

**FIGURE 2:**
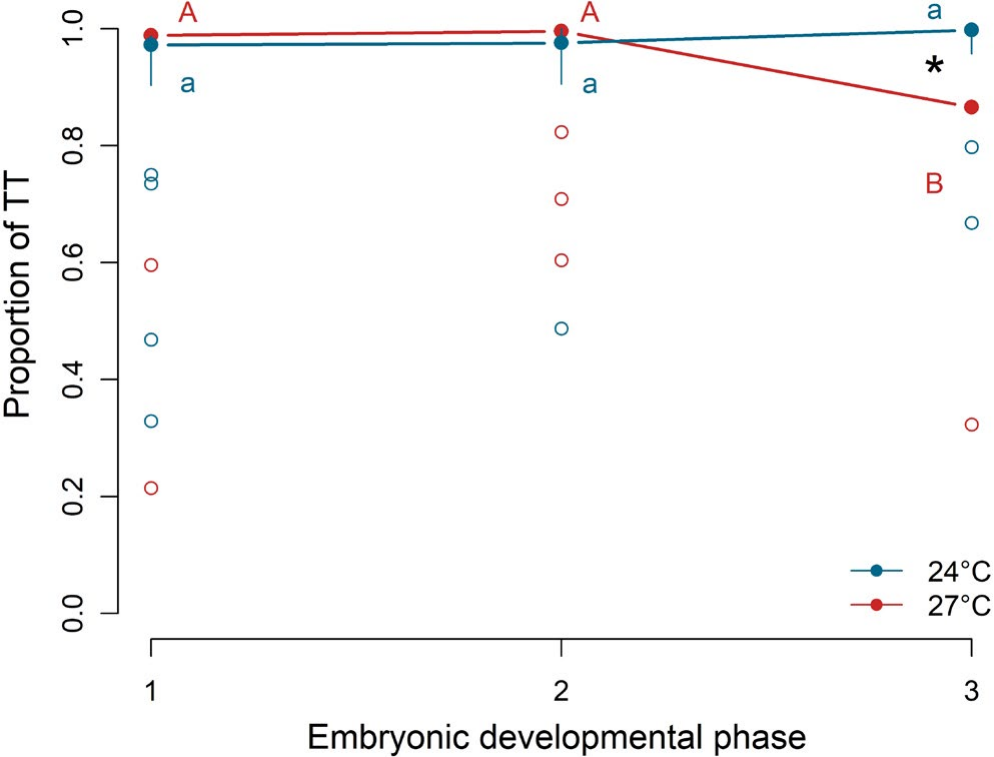
Relationship between phase of embryonic development and the proportion of the total time (TT) spent by parents carrying out parental care activity in the marine annelid *O. labronica* measured at 24 (blue dots and blue line) and 27℃ (red dots and red line). Solid dots represent the median, top and bottom vertical whiskers represent quartiles, and empty dots indicate outliers. Capital and lower‐case letters represent significant differences (*p* < .05) between different phases of the embryonic development for the elevated and control temperature conditions, respectively. Asterisk (*) indicates significant differences (*p* < .05) between temperature conditions within the same phase of embryonic development

The proportion of total time for parental care activity (TT) ranged between 0.83 ± 0.04 (mean ± *SE*) at 27℃ and 0.95 ± 0.02 at 24℃, in Phase 3 (Figure 2). Only in Phase 3, the proportion of TT was significantly lower for pairs reared at 27℃ compared with those at 24℃ (*t*_(96)_ = 3.26; *p* = .02; Table 2 and Figure 2), while it was comparable in Phase 1 and 2 (*p* > .05; Table 2 and Figure 2). Differences in the proportion of TT during different phases of embryonic development within the same temperature condition were observed only at 27℃, more specifically between Phase 2 and 3 (*t*_(96)_ = 3.72; *p* = .004) and between Phase 1 and 3 (*t*_(96)_ = 3.06; *p* = .03) (Figure 2; Table S4 in Appendix S3). No differences in the proportion of TT were found between phases of embryonic development at 24℃.

**TABLE 2:** Summary of statistical analyses for the effect of temperature (Temp) on the proportion of the total time spent for parental care activity (TT), relative contribution of the proportion of TT on hatching success, proportion of the total times spent for parental care activity by the female (TF) and male (TM) in isolation and simultaneously (TS) in the marine annelid *O. labronica*

			*df*	χ^2^		*p*
Parental care activity for TT						
Proportion of TT	Temp		1	0.01		.914
	Phase		2	3.62		.164
	Temp * Phase		**2**	**16.67**		**.0002**
			Phase 1 (*t* = −1.93; *p* = .39) Phase 2 (*t* = −1.52; *p* = .65) **Phase 3 (*t* = 3.26; *p* = .019)**			
Hatching success						
	Temp		**1**	**5.58**		**.018**
	TT		1	0.52		.471
	Temp * TT		1	1.94		.163
Parental care activity for TF, TM, and TS						
Proportion of TF	Temp	1		0.01		.930
	Phase	2		2.58		.275
	Temp * Phase	**2**		**10.53**		**.005**
		Phase 1 (*t* = 1.03; *p* = .908) Phase 2 (*t* = −2.57; *p* = .539) Phase 3 (*t* = −1.33; *p* = .115)				
Proportion of TM	Temp	1		0.02		.879
	Phase	**2**		**8.19**		**.017**
	Temp * Phase	**2**		**18.50**		**< .0001**
		Phase 1 (*t* = 2.50; *p* = .134) Phase 2 (*t* = 0.67; *p* = .985) **Phase 3 (*t* = −3.44; *p* = .011)**				
Proportion of TS	Temp	1			0.02	.892
	Phase	**2**			**8.19**	**.012**
	Temp * Phase	**2**			**18.50**	**< .0001**
		Phase 1 (*t* = 2.77; *p* = .071) Phase 2 (*t* = 1.15; *p* = .858) **Phase 3 (*t* = −4.16; *p* = .001)**				

Note

Only comparisons between the two temperature conditions within each phase of embryonic development are reported for the analysis of the proportion of TT, TF, TM, and TS (see Tables S4 and S5 in Appendix S3 for all pairwise comparisons). Degree of freedom (*df*), Wald Chi‐squared (χ^2^), and probability levels (*p*) are provided. Significant effects are reported in bold and the results of pairwise contrasts are indicated within brackets.

### 3.2 Effect of temperature and parental care activity on reproductive success

**FIGURE 3:**
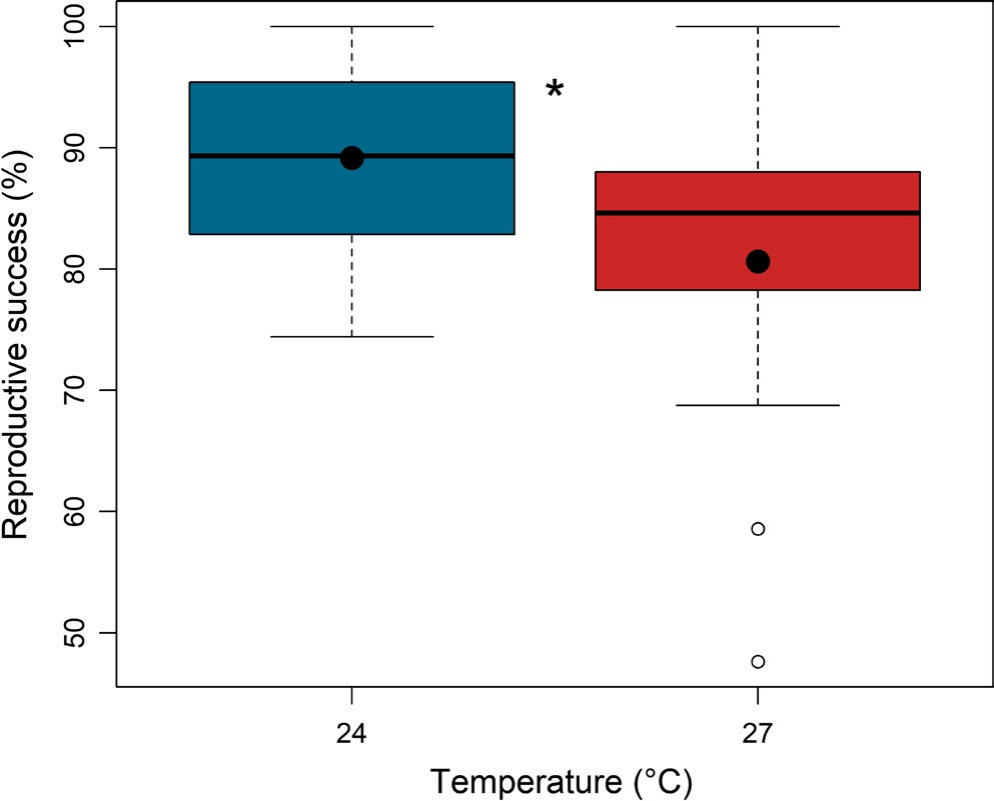
Effect of temperature on the reproductive success of *O. labronica*. Mean values of the reproductive success (%) at 24℃ and 27℃ are reported as black dots. The median (horizontal dark line in each box), quartiles (top and bottom of box), and the extreme of the lower and upper whiskers are shown for each group. Empty dots indicate outliers. An asterisk (*) indicates significant differences (*p* < .05) between temperature conditions

Hatching success decreased significantly from 89% to 81% at 24℃ and at 27℃, respectively (Figure 3). Temperature was the only factor significantly affecting hatching success (χ^2^_(1)_ = 5.58; *p* = .02; Table 2), while Total TT and its interaction with temperature did not have any significant effect on this trait (Table 2).

### 3.3 Effect of temperature on the sex‐related division of parental care

**FIGURE 4:**
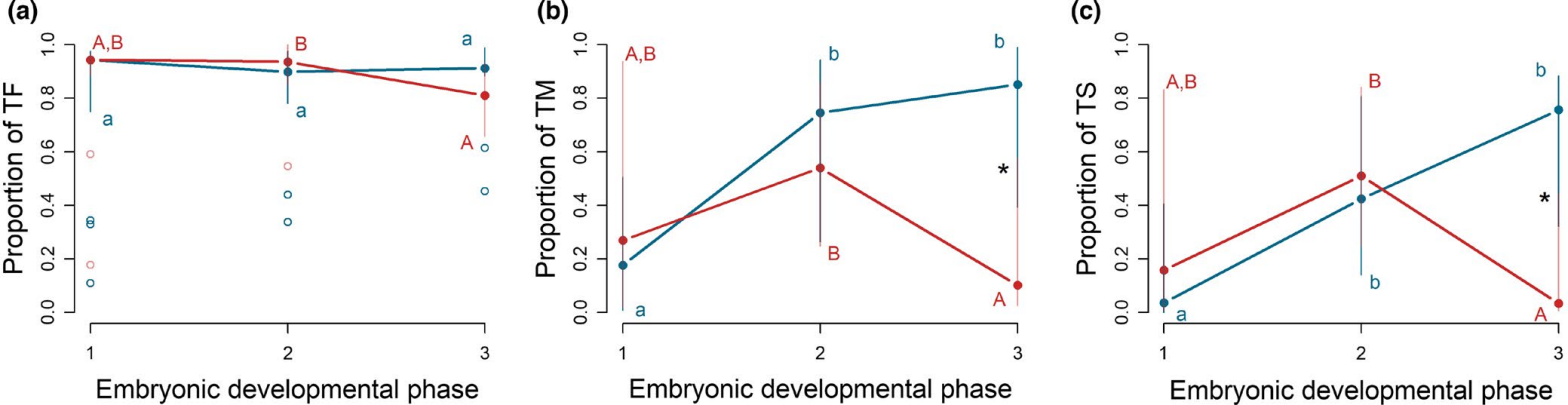
Relationship between phase of embryonic development and time spent by (a) the female (TF), (b) the male (TM), and both parents simultaneously (TS) of *O. labronica* carrying out parental care activity at 24 (blue) and 27℃ (red). Solid dots represent the median, top and bottom vertical whiskers represent quartiles, and empty dots indicate outliers. Capital and lowercase letters represent significant differences (*p* < .05) between phases of embryonic development for the elevated and control temperature conditions, respectively. Asterisks (*) indicate significant differences (*p* < .05) between temperature conditions within the same phase of embryonic development

Overall, the proportion of the time spent carrying out parental cares separately by the female (TF), male (TM), and the two partners simultaneously (TS) significantly change along the different phases of embryonic development depending on the temperature conditions tested, as shown by the presence of significant interactions between “Temp” and “Phase” (TF: *p* < .01; TM, *p* < .001; S, *p* < .001) (Table 2; Table S5 in Appendix S3 for the pairwise results). In detail, a significant decrease in the proportion of TF was observed between Phase 2 and 3 at 27℃ (*t*_(96)_ = 3.21; *p* = .02), while no differences were found at 24℃ (Figure 4a; Table S5 in Appendix S3). The proportion of TF within a given phase of egg development did not differ with temperature (*p* > .05; Table 2; Figure 4a).

The proportion of TM was significantly lower at 27℃ (0.3 ± 0.08; mean ±SE) than 24℃ (0.69 ± 0.09) in the third phase of egg development (*t*_(96)_ = −3.44; *p* = .01; Table 1; Figure 4b). In addition, at 27°C, this trait was significantly lower at Phase 3 compared with Phase 2 (*t*_(96)_ = 2.98; *p* = .04), while trait values at Phase 1 and Phase 3 were comparable (Figure 4b; Table S5 in Appendix S3). Contrarily, the proportion of TM increased significantly from Phase 1 to Phase 2 (*t*_(96)_ = −2.94; *p* = .046), as well as from Phase 1 to Phase 3 (*t*_(96)_ = −4.07; *p* = .001) at 24℃.

Finally, the proportion of TS was significantly lower at 27℃ (0.21 ± 0.07) when compared to 24℃ (0.62 ± 0.09), but only at Phase 3 (*t*_(96)_ = −4.16; *p* = .001; Table 2; Figure 4c). In addition, it decreased significantly from Phase 2 to Phase 3 (*t*_(96)_ = 3.79; *p* = .003) at 27℃, while was comparable between Phase 1 and 2, as well as between Phase 1 and 3 (Figure 4c; Table S5 in Appendix S3). By contrast, the proportion of TS showed the tendency to increase significantly from Phase 1 to Phase 2 (*t*_(96)_ = −2.92; *p* = .049) and from Phase 1 to Phase 3 at 24℃ (*t*_(96)_ = −4.44; *p* = .0003), Phase 2 and 3 showing comparable results.

## 4 DISCUSSION

Our study is one among the few investigating thermal plasticity in parental care behaviors in marine invertebrates with biparental care systems, and its role in affecting organisms' reproductive success within a climate change context. Whether organisms will be able to adjust or adapt to ongoing ocean warming is a central question in global change biology (Calosi et al., 2016; Chakravarti et al., 2016; Donelson et al., 2018; Shama, 2015). Behavioral plasticity provides an organism with an immediate tactical response to rapidly changing conditions, thus representing the first barrier of defense against the negative impacts of climate changes (Kearney et al., 2009; Sih et al., 2011; Walther et al., 2002).

Here we show that, in the marine annelid *Ophryotrocha labronica*, exposure to an elevated temperature can reduce the total time spent by parents caring for their brood, as well as the time of simultaneous parental care, during the last phase of embryonic development. These responses seem to be driven by a reduction in the time spent by the male in performing parental care activities in the third phase. Interestingly, this behavioral plasticity is not related to the parents' short‐term fitness, measured as hatching success, despite the fact that the latter was negatively affected by the exposure to the elevated temperature tested.

The reduction in parental care observed only during the third phase of development at the highest temperature tested may be explained by the existence of cost‐benefit trade‐offs between the parental care investment and offspring fitness (Winkler, 1987). Evolutionary theory on parental care predicts that selection favors the evolution of parental care strategies when the costs of providing care (e.g., higher energetic demand, reduced parental survival, or future reproduction) do not outweigh its benefits (i.e., higher offspring survival and quality) (Clutton‐Brock, 1991; Klug & Bonsall, 2014; Pike & Wen‐san Huang, 2013; Winkler, 1987). Accordingly, organisms may have evolved multiple behavioral responses able to guarantee that the overall beneficial nature of their parental care strategy is maintained also under stressful conditions, such as rapid thermal changes. In *Ophryotrocha labronica*, this condition may have been achieved through the fine‐tuning of parental care behaviors during embryo development. For example, by evolving temperature‐independent parental care behaviors at those stages of embryonic development, specifically from cleavage to gastrulation, that in some marine invertebrates are more vulnerable to the negative effects of temperature (Andronikov, 1975; Cossins & Bowler, 1987; Kinne & Kinne, 2011), that is, the first and second phase in *O. labronica*. Therefore, by evolving a less strict tie with the offspring at a given stage, that is, the third phase, when embryos are more developed and able to actively move inside the egg mass case. The latter strategy may allow for a reduction in parental care investment at the elevated temperature, enabling parents to cope with the increased energetic demand they incur in, without negatively affecting offspring's survival. From a mechanistic perspective, the greater energetic demand commonly experienced by ectotherms at higher temperatures as a result of increased cell kinetics (Angilletta, 2009; Hochachka & Somero, 2002) may be the consequence of having to allocate more energy to fuel cell maintenance, repair, and other costly whole‐organism functions (Schaffer, 1974; Stearns, 1992). This increased cost may be likely sustained at 27℃ during the third phase of embryonic development in *O. labronica*, or cumulatively up to this phase. On the contrary, reproductive performance and parental care activities at the control condition may have not resulted in additional costs associated with parental investment and, consequently, in the necessity to alter parental care behavior along the eggs' development.

Several studies on aquatic ectotherms have shown a negative correlation between time spent by parents caring for their offspring versus parental investment in self‐maintenance. Marconato et al. (1993), for example, found that somatic conditions (body weight) of males of the river bullhead *Cottus gobio* (Linnaeus, 1758) declined proportionally with the time spent undertaking parental care activities. Similarly, in the marine mantis shrimp *Pullosquilla thomassini* (Manning, 1978), a species exhibiting biparental care, a reduction in body mass was detected as a consequence of increased parental activity of the male partner, probably to compensate for the absence of the other caregiver (Wright & Caldwell, 2015). In our study, the lack of changes either in growth rate or body size at maturity of parents due to a temperature increase—although we could not estimate other metrics of body condition—suggests that *O. labronica* may have the ability to release energy for somatic maintenance that benefits current adult performance at the advantage of future reproduction (Martins & Wright, 1993; Roff, 2002), ultimately maximizing parental fitness on a longer term under the novel thermal condition (Nagelkerken & Munday, 2016). In our study, we are unable to demonstrate the existence of the trade‐off between short‐term and longer‐term fitness, as well as its relationship with thermal plasticity in parental care behaviors. However, we know that the first reproductive events (1–3) provide the greatest contribution in defining the population growth rate of *O. labronica* at high temperatures (Prevedelli & Simonini, 2001). Given the positive relationship commonly found between female body size and fecundity in this species (Berglund, 1991; Prevedelli et al., 2006; Thornhill et al., 2009), a relatively higher investment in self‐maintenance under increasing temperatures may increase chances for longer‐term fitness, and thus indirectly result in greater fitness at the population level.

The production of smaller broods may have favored the reduction in parental care investment. In many marine invertebrates, in fact, larger brood contains a higher proportion of eggs located deep in the clutch, thus requiring more ventilation in order for oxygen to reach the center of the egg mass (Baeza & Fernández, 2002; Cohen & Strathmann, 1996; Fernández et al., 2000; Strathmann & Strathmann, 1995). Accordingly, a smaller amount of eggs in the clutch, as observed in *O. labronica* at the elevated temperature, would require less care, thus allowing parents to preserve energy for self‐maintenance, repair, growth, and future reproductive investments, as postulated by the *Parental Investment Theory* (Sargent & Gross, 1986; Williams, 1966). On the contrary, the increase in parental investment when broods are larger can be explained by the increased fitness value that larger broods represent (Galvani & Coleman, 1998). In our study, we indeed find a positive relationship between brood size and all four measurements of parental effort, a result that is consistent with experimental observations showing an increase in maternal and paternal care investment in larger broods in the congeneric hermaphroditic annelid *Ophryotrocha diadema (*Åkesson, 1976) (Picchi & Lorenzi, 2019).

Interestingly, the proportion of total time spent by parents in caring for their offspring in *O. labronica* does not increase with the progression of embryonic development, as documented in other marine ectotherms as a strategy to sustain the higher energetic demand of growing embryos (Baeza & Fernández, 2002; Green & McCormick, 2005). Neither we observe an overall trend of decreasing parental care activity across developmental stages as found in other aquatic species, where embryos gained the ability to self‐ventilate toward the end of development (Dick et al., 1998). This variety of responses suggests that more than one strategy exists in marine invertebrates for parental care investments across development.

Regarding our second research aim, we did not find any significant relationship between the time spent for parental care activity and the hatching success under the elevated temperature condition. We report a moderate, but significant, 7% reduction in reproductive success compared with the control condition. However, this change is not related to the thermal plasticity of parental care activity observed in response to exposure to an elevated temperature. Hopkins et al. (2011) reported that a negative effect of elevated temperature on reproductive success was accompanied by an increase in parental activity in the three‐spined stickleback *Gasterosteus aculeatus* (Linnaeus 1758), but the authors did not formally test for the presence of a relationship between these two traits. Similarly, to our study, no apparent relationship between reproductive success and total parental care at elevated temperature was observed in the burying beetle *Nicrophorus orbicollis* (Fabricius, 1775) (Ong, 2019). Therefore, hatching success may be independent from limited changes in the amount of care embryos receive from the parents.

Finally, we found a sex‐related contribution to the care of the eggs at the elevated temperature. In particular, males' parental investment was less than a half of that provided by females in the last phase of egg development at 27℃, when compared to our control conditions when male contribution represented 20% of that of the female. The existence of sex‐specific behavioral patterns in the genus *Ophryotrocha* was also demonstrated by Picchi and Lorenzi (2019), who found that parental care was a female‐biased behavior both in *O. labronica* and the hermaphroditic *O. diadema*. In addition, they observed that males were less constrained by parental duties and invested more effort (e.g., increased motility) to increase mating opportunities, especially at higher densities (Picchi & Lorenzi, 2019). We may conclude that sex‐biased plasticity can also be induced by factors other than density, such as conditions of thermal stress tested in our study. More in general, our results are in line with several studies, almost exclusively conducted on birds, showing that increased temperatures affected investment patterns in species with biparental care, with the dominant protector (i.e., the female in our study) and the subordinate one (i.e., the male) responding differently to this environmental challenge (Wiley & Ridley, 2016; Vincze et al., 2017). In addition, females' parental investment is neither affected by temperature or by the reduced males' parental care under elevated temperature. This is in accordance with the “No negotiation” model, according to which one parent alters its investment in the offspring independently from the level of investment of its partner (Houston, 1985). The absence of a negotiation strategy in this species may be due to the differences in costs and benefits of parental care between sexes. In fact, males of this species appear to have much more fitness advantages by engaging in multiple mating events than undertaking parental care activity, while for females it appears more advantageous to maintain parental care investment to maximize their fitness (Picchi & Lorenzi, 2019). Altogether, the significant reduction in males' care activities and the simultaneous contribution of males and females to parental care activities appear to be responsible for the general decrease in total parental care activity during the late phase of embryonic development at elevated temperatures. Manipulative experiments, monitoring parental care behaviors of one parent in response to the removal of its partner, would help to more definitively confirm the existence of these patterns of biparental care in this species under elevated temperature.

In summary, our findings showed that ocean warming will exert negative effects on the reproductive success of *O. labronica*. However, this species appears to have evolved a parental care strategy that enables it to maintain a positive cost‐benefit trade‐off between parents and offspring, with potential benefits for parents' individual and species fitness (e.g., successive reproductive events) under elevated temperatures. This suggests that plasticity in parental care behavior is a mechanism that can partially mitigate the negative effects of temperature‐dependent impacts; however, how this mechanism will play out along the life span of individuals, and thus contribute to population level responses in the longer term, is still to be determined. Nonetheless, our results contribute to the ongoing debate on the role and limits of behavioral plasticity as a coping strategy to buffer the impact of rapid environmental change.

## CONFLICT OF INTEREST

None declared.

## AUTHOR CONTRIBUTIONS

**Davide Spatafora**: Conceptualization (Lead); data curation (lead); formal analysis (lead); funding acquisition (equal); investigation (lead); methodology (lead); project administration (equal); writing—original draft (lead). **Gloria Massamba N'Siala**: Conceptualization (supporting); data curation (supporting); formal analysis (supporting); investigation (supporting); methodology (supporting). **Federico Quattrocchi**: Data curation (Supporting); formal analysis (supporting); investigation (supporting); methodology (supporting). **Marco Milazzo**: Conceptualization (Equal); data curation (equal); formal analysis (equal); funding acquisition (equal); investigation (equal); methodology (equal); project administration (equal). **Piero Calosi**: Conceptualization (Equal); data curation (equal); formal analysis (equal); funding acquisition (equal); investigation (equal); methodology (equal); project administration (equal).

## Supporting information

Appendix S1‐S3

## Data Availability

The datasets generated for this study are deposited in Dryad digital repository at https://doi.org/10.5061/dryad.m0cfxpp47.
