# Supplementary material for: Plastic adjustments of biparental care behavior across embryonic development under elevated temperature in a marine ectotherm
Source: Ecol Evol. 2021 Jul 29;11(16):11155–67. doi: 10.1002/ece3.7902 (PMC8366872; doi:10.1002/ece3.7902)
Supplement: Supplementary file 1 — Appendix S1‐S3 [file ECE3-11-11155-s001.docx]

**SUPPORTING INFORMATION**

**Title:** **Plastic adjustments of biparental care behaviour across embryonic development and under elevated temperature in a marine ectotherm**

Davide Spatafora, Gloria Massamba N’Siala, Federico Quattrocchi, Marco Milazzo, Piero Calosi

**APPENDIX S1**

**Effect of temperature on** **brood size, body size, and growth rate**

*Statistical analyses*

A set of preliminary analyses on a number of life-history traits (i.e. brood size, body size, and growth rate) was performed to support the interpretation of the results on the effect of temperature on parental care investment. Specifically, the combined effect of temperature and female body size, used as covariate, on brood size was analysed using a Poisson-GLM (P-GLM) corrected for overdispersion using quasi-Poisson GLM (QP GLM). P-GLM was also used to assess the effect of temperature on male and female body size. Similarly, a P-GLM with total days used as an offset variable was performed to assess the effect of temperature on the growth rate of both parents.

*Results*

Only brood size was significantly reduced under the elevated temperature (Table A1; Fig. A1a), while temperature had no effect on growth rate and body size of both parents (Table A1; Fig. A1b-e).

| **Table A1** Sequential analysis of deviance based on Wald chi-squared test for the effect of temperature (Temp) on different life-history traits (brood size, parents’ body size and growth rate) in the marine annelid *O. labronica*. Degree of freedom (df), Chisq (χ2) and probability levels (p) are provided (significant effects: p < 0.05). | | | | |
| --- | --- | --- | --- | --- |
| **LIFE HISTORY TRAITS** | |  |  |  |
|  | | **df** | **χ^2^** | **p** |
| **Brood size** | **Temp** | **1** | **8.06** | **0.005** |
|  | Female body size | 1 | 2.41 | 0.120 |
|  | Temp * Female body size | 1 | 2.31 | 0.128 |
|  | |  |  |  |
| Female body size | Temp | 1 | 0.002 | 0.964 |
|  | | | | |
| Male body size | Temp | 1 | 0.49 | 0.484 |
|  | | | | |
| Female growth rate | Temp | 1 | 0.39 | 0.531 |
|  |  |  |  |  |
| Male growth rate | Temp | 1 | 0.74 | 0.391 |


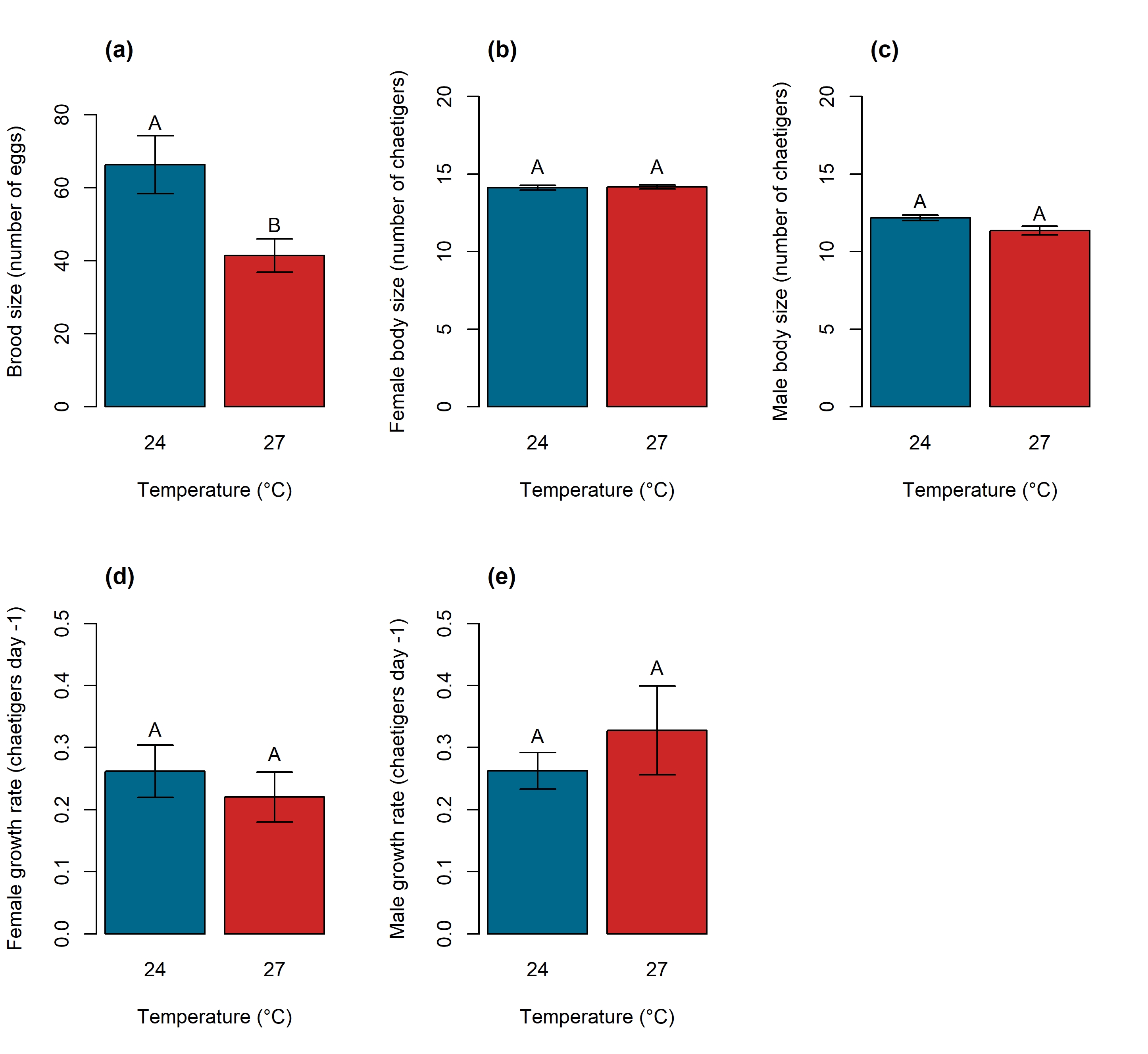


**Figure A1** Effect of temperature on (a) brood size, (b) female body size, (c) male body size, (d) female growth rate, and (e) male growth rate in the marine annelid *O. labronica*. Mean (±S.E.) are reported. Different capital letters indicate significant differences between temperature conditions (α = 0.05).

**APPENDIX S2**

**Relation between parents growth rate, brood size and parental care behaviour**

*Statistical analyses*

Preliminary analyses were performed to assess the role of parental growth rate and brood size in affecting the proportional time of parental care. In particular, to test the relationships between parental growth rate (log transformed) and the proportion of parental care time provided by each sex (defined TF for the female and TM for the male) in each embryo developmental phase (“Phase” – fix factor with three levels: Phase 1, 2, 3) at the two temperature conditions (“Temp” – fix factor with two levels: 24 and 27°C), we used a linear model test, separately for each sex.

The relationship between the proportion of total time for parental care activity (TT) and brood size was tested using a binomial generalized mixed model (B-GLMM) with lme4 package for R (Bates et al., 2015). Similarly, three B-GLMMs were used to test the proportion of the time spent carrying out parental cares by each descriptor (TF, TM and TS) and brood size. The identity of the experimental pairs (“Pair” –factor with thirty-six levels: pair 1 to 36) was used as random factor (random intercept model), as observations were repeated on the same pair along the three phases of embryonic development, thus violating the assumption of independence.

*Results*

No significant effect of parental care on both female and males’ growth rate were observed (Table A2). In addition, we found that the proportion of TT, the proportion of TF and TM decreased significantly with the reduction of the brood size (Table A3; Fig. A2)

| **Table A2** Parameters estimation of the linear regressions ran to test the effect of the proportion of time spent by single parents for parental care (TF and TM) on both female and males’ growth rate at the control and elevated temperature condition in the marine annelid *O. labronica*. Estimate (Est.), Standard Error (S.E.), t-value (t) and probability levels (p) are provided (significant effects: p < 0.05). | | | | | |
| --- | --- | --- | --- | --- | --- |
|  | | **Est.** | **S.E.** | **t** | **p** |
| **Females’ growth rate** | (Intercept) | -0.1249 | 0.3978 | -0.314 | 0.7561 |
|  | Phase 1 | 0.1109 | 0.1723 | 0.644 | 0.5253 |
|  | Phase 2 | -0.2649 | 0.2295 | -1.155 | 0.2587 |
|  | Phase 3 | 0.5194 | 0.3837 | 1.354 | 0.1875 |
|  | 27°C | 0.6977 | 0.4989 | 1.398 | 0.1738 |
|  | Phase 1 * 27°C | 0.6481 | 0.3452 | 1.878 | 0.0717 |
|  | Phase 2 * 27°C | -0.8383 | 0.5186 | -1.616 | 0.1181 |
|  | Phase 3 * 27°C | -0.564 | 0.4352 | -1.296 | 0.2064 |
| **Males’ growth rate** | (Intercept) | 0.2017235 | 0.6239449 | 0.323 | 0.749 |
|  | Phase 1 | -0.0134682 | 0.2701731 | -0.05 | 0.961 |
|  | Phase 2 | -0.0006363 | 0.3598707 | -0.002 | 0.999 |
|  | Phase 3 | 0.0415466 | 0.6018673 | 0.069 | 0.945 |
|  | 27°C | -0.5081616 | 0.7824254 | -0.649 | 0.522 |
|  | Phase 1 * 27°C | -0.2988153 | 0.5413387 | -0.552 | 0.586 |
|  | Phase 2 * 27°C | 0.6428327 | 0.8134289 | 0.79 | 0.437 |
|  | Phase 3 * 27°C | 0.25699 | 0.6826344 | 0.376 | 0.71 |

| **Table A3** Anova results for the relationship between the proportion of the total time for parental care activity (TT) (a), separate and simultaneous contribution of female and male to parental care activity (TF, TM and TS respectively) (b-d), and brood size in the marine annelid *O. labronica*. Degree of freedom (df), Wald Chisq (χ2) and probability levels (p) are provided (significant effects: p < 0.05). | | | | |
| --- | --- | --- | --- | --- |
|  |  | **df** | **χ^2^** | ***p*** |
| **(a) Proportion of TT** | | 1 | 24.99 | **< 0.0001** |
| **(b) Proportion of TF** | | 1 | 17.644 | **< 0.0001** |
| **(c) Proportion of TM** | | 1 | 10.99 | **0.0009** |
| **(d) Proportion of TS** | | 1 | 12.16 | **0.0005** |


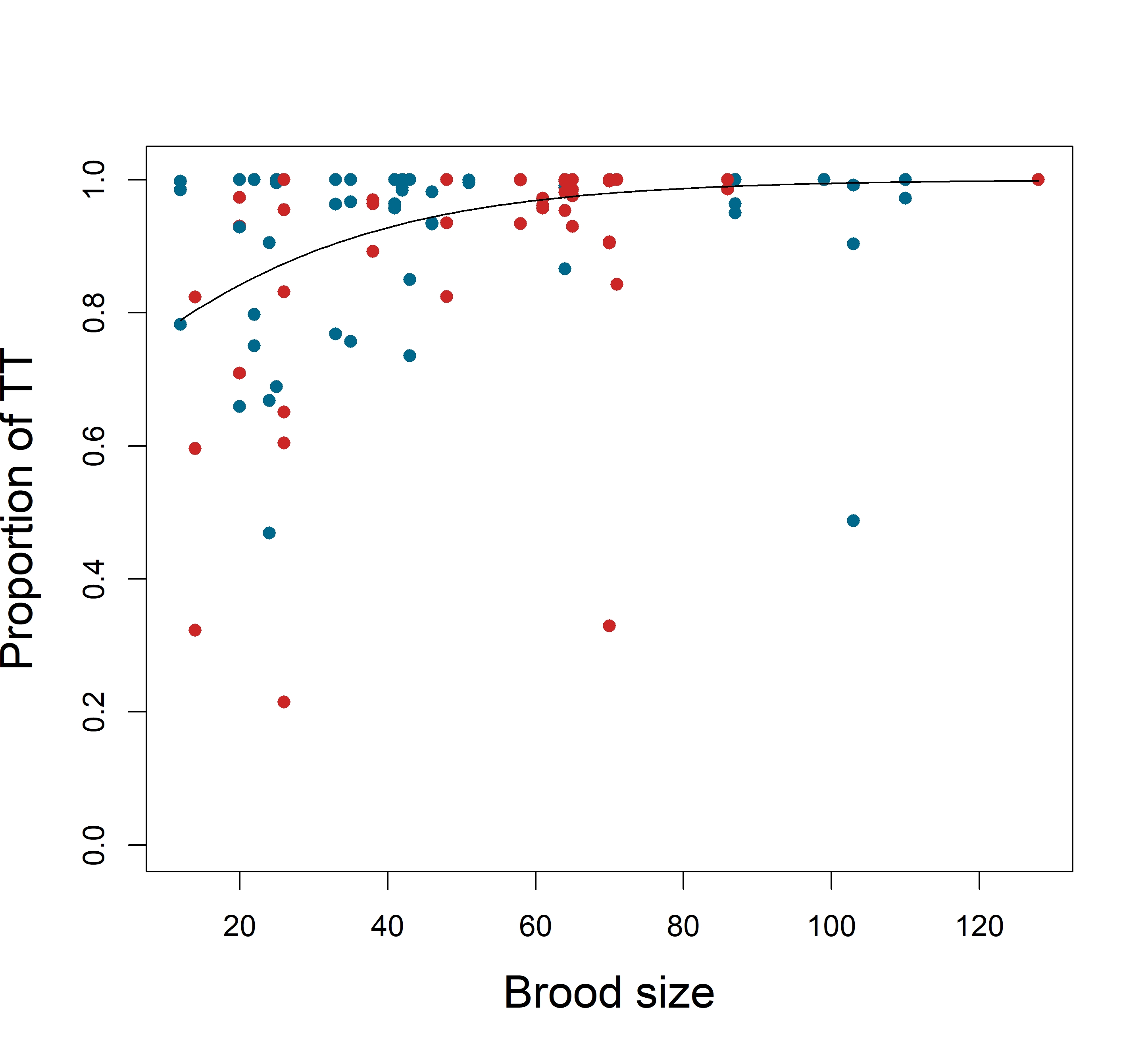


Figure A2 Relationship between brood size and proportion of the total time (TT) spent by individuals of the marine annelid *O. labronica*, carrying out parental care activity measured at 24 (blue dots) and 27°C (red dots). The black line represents the fitted value of the glmm model.

**APPENDIX S3**

**Effect of temperature on parental care activity (pairwise comparisons)**

| **Table A4** Pair-wise results for the significant interaction between temperature and phase of embryonic development for the proportion of the total time spent for parental care activity in the marine annelid *O. labronica*. Degree of freedom (df), t-ratio(t) and probability levels (p) are provided and significant effects (p < 0.05). Significance levels are based on the adjusted p-values from Tukey’s HSD. | | | | |
| --- | --- | --- | --- | --- |
| **Total time Parental care** |  | **df** | ***t*** | **p** |
| **Within Temperature** | **24°C** |  |  |  |
|  | Phase 1 – Phase 2 | 96 | -1.07 | 0.893 |
|  | Phase 2 – Phase 3 | 96 | -1.07 | 0.892 |
|  | Phase 1 – Phase 3 | 96 | -2.14 | 0.277 |
|  | **27°C** |  |  |  |
|  | Phase 1 – Phase 2 | 96 | -0.66 | 0.986 |
|  | **Phase 2 – Phase 3** | **96** | **3.72** | **0.004** |
|  | **Phase 1 – Phase 3** | **96** | **3.06** | **0.034** |
| **Within Phase** | **Phase 1** | 96 |  |  |
|  | 27°C – 24°C | 96 | -1.93 | 0.392 |
|  | **Phase 2** | 96 |  |  |
|  | 27°C – 24°C | 96 | -1.52 | 0.650 |
|  | **Phase 3** | 96 |  |  |
|  | **27°C – 24°C** | **96** | **3.26** | **0.019** |
| **Other contrasts** | 27°C, Phase 1 – 24°C, Phase 2 | 96 | -0.86 | 0.955 |
|  | 27°C, Phase 1 – 24°C, Phase 3 | 96 | 0.21 | 0.999 |
|  | 27°C, Phase 2 – 24°C, Phase 3 | 96 | -0.45 | 0.998 |
|  | 24°C, Phase 1 – 27°C, Phase 2 | 96 | -2.59 | 0.110 |
|  | 24°C, Phase 1 – 27°C, Phase 3 | 96 | 1.13 | 0.869 |
|  | 24°C, Phase 2 – 27°C, Phase 3 | 96 | 2.20 | 0.250 |

|  | **Table A5** Results for the pair-wise comparison for significant interactions between the terms “Temp” and “Phase” for the proportion of the total times spent for parental care activity by the female (TF) and male (TM) in isolation and simultaneously (TS) in the marine annelid *O. labronica*. Degree of freedom (df), t-ratio (t), probability levels (p) are provided and significant effects (p < 0.05). Significance levels are based on the adjusted p-values from Tukey’s HSD. | | | | | |
| --- | --- | --- | --- | --- | --- | --- |
| **PARENTAL CARE ACTIVITY FOR TF, TM, AND S** | | |  | **df** | **t** | **p** |
|  | | |  |  |  |  |
| **Proportion of TF** | | **Within Temperature** | **24°C** |  |  |  |
|  |  |  | Phase 1 – Phase 2 | 96 | 0.10 | 1 |
|  |  |  | Phase 2 – Phase 3 | 96 | -1.05 | 0.898 |
|  |  |  | Phase 1 – Phase 3 | 96 | -0.95 | 0.931 |
|  |  |  |  |  |  |  |
|  |  |  | **27°C** |  |  |  |
|  |  |  | Phase 1 – Phase 2 | 96 | -0.57 | 0.993 |
|  |  |  | **Phase 2 – Phase 3** | **96** | **3.21** | **0.022** |
|  |  |  | Phase 1 – Phase 3 | 96 | 2.65 | 0.097 |
|  |  | **Within Phase** | **Phase 1** |  |  |  |
|  |  |  | 27°C – 24 °C | 96 | 1.03 | 0.908 |
|  |  |  | **Phase 2** |  |  |  |
|  |  |  | 27°C – 24°C | 96 | 1.69 | 0.539 |
|  |  |  | **Phase 3** |  |  |  |
|  |  |  | 27°C – 24°C | 96 | -2.57 | 0.115 |
|  |  | **Other contrasts** | 27°C, Phase 1 – 24°C, Phase 2 | 96 | 1.13 | 0.868 |
|  |  |  | 27°C, Phase 2 – 24°C, Phase 3 | 96 | 0.64 | 0.988 |
|  |  |  | 27°C, Phase 1 – 24°C, Phase 3 | 96 | 0.07 | 1 |
|  |  |  |  |  |  |  |
|  |  |  | 24°C, Phase 1 – 27°C, Phase 2 | 96 | 1.59 | 0.604 |
|  |  |  | 24°C, Phase 2 – 27°C, Phase 3 | 96 | -1.52 | 0.654 |
|  |  |  | 24°C, Phase 1 – 27°C, Phase 3 | 96 | -1.62 | 0.589 |
| **Proportion of TM** | | **Within Temperature** | **24°C** |  |  |  |
|  |  |  | **Phase 1 – Phase 2** | **96** | **-2.94** | **0.046** |
|  |  |  | Phase 2 – Phase 3 | 96 | -1.13 | 0.868 |
|  |  |  | **Phase 1 – Phase 3** | **96** | **-4.07** | **0.001** |
|  |  |  | **27°C** |  |  |  |
|  |  |  | Phase 1 – Phase 2 | 96 | -1.11 | 0.877 |
|  |  |  | **Phase 2 – Phase 3** | **96** | **2.98** | **0.041** |
|  |  |  | Phase 1 – Phase 3 | 96 | 1.87 | 0.425 |
|  |  | **Within Phase** | **Phase 1** |  |  |  |
|  |  |  | 27°C – 24°C | 96 | 2.50 | 0.134 |
|  |  |  | **Phase 2** |  |  |  |
|  |  |  | 27°C – 24°C | 96 | 0.67 | 0.985 |
|  |  |  | **Phase 3** |  |  |  |
|  |  |  | **27°C – 24°C** | **96** | **-3.44** | **0.011** |
|  |  | **Other interactions** | 27°C, Phase 1 – 24°C, Phase 2 | 96 | -0.44 | 0.9980 |
|  |  |  | 27°C, Phase 2 – 24°C, Phase 3 | 96 | -0.46 | 0.9975 |
|  |  |  | 27°C, Phase 1 – 24°C, Phase 3 | 96 | -1.56 | 0.6242 |
|  |  |  |  |  |  |  |
|  |  |  | **24°C, Phase 1 – 27°C, Phase 2** | **96** | **3.61** | **0.0063** |
|  |  |  | 24°C, Phase 2 – 27°C, Phase 3 | 96 | -2.31 | 0.2009 |
|  |  |  | 24°C, Phase 1 – 27°C, Phase 3 | 96 | 0.63 | 0.9887 |
| **Proportion of TS** | | **Within Temperature** | **24°C** |  |  |  |
|  |  |  | **Phase 1 – Phase 2** | **96** | **-2.92** | **0.049** |
|  |  |  | Phase 2 – Phase 3 | 96 | -1.52 | 0.656 |
|  |  |  | **Phase 1 – Phase 3** | **96** | **-4.44** | **0.0003** |
|  |  |  | **27°C** |  |  |  |
|  |  |  | Phase 1 – Phase 2 | 96 | -1.29 | 0.786 |
|  |  |  | **Phase 2 – Phase 3** | **96** | **3.79** | **0.003** |
|  |  |  | Phase 1 – Phase 3 | 96 | 2.50 | 0.134 |
|  |  | **Within Phase** | **Phase 1** |  |  |  |
|  |  |  | 27°C – 24°C | 96 | 2.77 | 0.071 |
|  |  |  | **Phase 2** |  |  |  |
|  |  |  | 27°C – 24°C | 96 | 1.15 | 0.858 |
|  |  |  | **Phase 3** |  |  |  |
|  |  |  | **27°C – 24°C** | **96** | **-4.16** | **0.001** |
|  |  | **Other interactions** | 27°C, Phase 1 – 24°C, Phase 2 | 96 | -0.15 | 1 |
|  |  |  | 27°C, Phase 2 – 24°C, Phase 3 | 96 | -0.36 | 0.999 |
|  |  |  | 27°C, Phase 1 – 24°C, Phase 3 | 96 | -1.66 | 0.561 |
|  |  |  |  |  |  |  |
|  |  |  | **24°C, Phase 1 – 27°C, Phase 2** | **96** | **4.07** | **0.001** |
|  |  |  | 24°C, Phase 2 – 27°C, Phase 3 | 96 | -2.65 | 0.096 |
|  |  |  | 24°C, Phase 1 – 27°C, Phase 3 | 96 | 0.27 | 1 |

**Reference:**

Bates, D., Mächler, M., Bolker, B., & Walker, S. (2015). Fitting linear mixed‐effects models using lme4. Journal of Statistical Software, 67, 1–48.
